# LINC02086 promotes cell viability and inhibits cell apoptosis in breast cancer by sponging miR-6757-5p and up-regulating EPHA2

**DOI:** 10.1186/s12957-023-03245-w

**Published:** 2023-11-27

**Authors:** Xue Han, Fan Shi, Shujun Guo, Yao Li, Hongtao Wang, Chuanwang Song, Shiwu Wu

**Affiliations:** 1 Department of Immunology, School of Laboratory Medicine, Bengbu Medical College, Bengbu, 233030 China; 2 Anhui Provincial Key Laboratory of Infection and Immunity, Bengbu Medical College, Bengbu, 233030 China; 3 Anhui Province Key Laboratory of Immunology in Chronic Diseases, Bengbu Medical College, Bengbu, 233030 China; 4 Department of Pathology, the First Affiliated Hospital of Bengbu Medical College, Bengbu, 233004 China; 5 Department of Pathology, Basic Medical College, Bengbu Medical College, Bengbu, 233030 China; 6 Department of Pathology, the Second People’s Hospital of Anhui Province, Hefei, 230041 China; 7 Key Laboratory of Cancer Translational Medicine Center of Anhui Province, Bengbu Medical College, Bengbu, 233030 China

**Keywords:** lncRNAs, miRNAs, EPHA2, Luciferase reporter assay, RNA pull-down assay, Breast cancer

## Abstract

**Background:**

Long non-coding RNAs (lncRNAs) are critical regulators in the initiation and progression of breast cancer. Our study aims to characterize the functions of LINC02086 which few published in breast cancer and decipher the downstream molecular mechanisms.

**Methods:**

LINC02086 expression is tested in RNA-seq data from GEPIA database, tumor tissue samples from hospital patients and breast cancer cell lines. LINC02086 was silenced or overexpressed by lenti-virus-mediated shRNAs, or pLVX-Puro plasmids. Luciferase reporter assay and RNA pull-down assay were applied to study interactions between LINC02086, miR-6757-5p and ephrin type-A receptor 2 (EPHA2). LINC02086-silencing MCF-7 cells were injected into mice to establish xenograft animal models.

**Results:**

Using RNA-seq data, tumor tissue samples and breast cancer cells, LINC02086 was consistently found to be up-regulated in breast cancer, and correlated with poorer prognosis. LINC02086 knockdown decreased cell viability, promoted cell apoptosis and suppressed tumor growth. LINC02086 interacted with miR-6757-5p that interacted with EPHA2.LINC02086 expression was negatively correlated with miR-6757-5p expression (*r* = -0.5698, *P* < 0.001) but was positively correlated with EPHA2 expression (*r* = 0.5061, *P* < 0.001). miR-6757-5p expression was negatively correlated with EPHA2 expression (*r* = -0.5919, *P* < 0.001). LINC02086 regulated EPHA2 via miR-6757-5p. miR-6757-5p/EPHA2 axis was a mediator of the effect of LINC02086 on cell viability and apoptosis.

**Conclusion:**

LINC02086 increases cell viability and decreases apoptotic cells in breast cancer by sponging miR-6757-5p to upregulate EPHA2. This study presents LINC02086/miR-6757-5p/EPHA2 axis as promising therapeutic targets for breast cancer intervention.

**Supplementary Information:**

The online version contains supplementary material available at 10.1186/s12957-023-03245-w.

## Introduction

Breast cancer is the most frequently diagnosed malignancy with the second highest cancer-related mortality rate in females worldwide [1, 2]. Breast cancer is morphologically and molecularly heterogeneous with distinctive clinic-pathological features and thus is classified into different biological subtypes [3, 4]. Despite the availability of multiple therapeutic approaches, outcome of metastatic breast cancer remains unfavorable [5]. Therefore, exploring molecular mechanisms may drive the development of individualized therapeutic regimens and improve clinical benefits for breast cancer patients.

Long non-coding RNAs (lncRNAs) have been acknowledged as multidimensional entities accountable for regulating cell fate and homeostasis [6]. LncRNAs-mediated regulatory networks are involved in the initiation and progression of breast cancer and present novel therapeutic targets and prognostic biomarkers [7]. LncRNAs can serve as sponges of microRNAs (miRNAs) and competitively bind to miRNAs to regulate mRNAs expression [8]. The current literature describes intricate interactions between lncRNAs and miRNAs, which exert tumor suppressor or oncogenic effects and have great promises as potential prognostic biomarkers and therapeutic targets for breast cancer [9, 10]. Integrative bioinformatics analyses uncover that LINC02086 promotes cells proliferation, invasion and migration of laryngeal squamous cell carcinoma (LSCC) and is related to survival of patients [11]. Moreover, LINC02086 is identified by bioinformatics analysis to be an important prognostic lncRNA in hepatocellular carcinoma patients with cirrhosis [12]. However, the functional roles and clinical significance of LINC02086 in breast cancer have not been dissected. Therefore, we used bioinformatics software (circinteractome) to search the downstream target miR-6757-5p of LINC02086. Up to now, there have been no relevant studies on miR-6757-5p in cancers.

Ephrin type-A receptor 2 (EPHA2) is predicted the target gene regulated bymiR-6757-5p. EPHA2 was observed in most epithelial cells from normal or tumor tissues. It is shown that EPHA2 is highly expressed in breast cancer [13]. Previous studies have found that EPHA2﻿ promotes breast cancer cell proliferation, migration, and invasion [14]. In addition, ﻿EPHA2 promotes breast cancer drug resistance [14]. However, the mechanism remains unclear.

The present study employed not only the downloaded RNA-seq data, but also tumor tissue specimens and human breast cancer cells lines to investigate LINC02086 expression in breast cancer. In vitro and in vivo experiments were used to elucidate the biological functions of LINC02086 in cell viability, cell apoptosis and tumor growth. Furthermore, sponge miRNAs, downstream target proteins and their biological activities were explored to decode the underlying molecular mechanisms of LINC02086 in breast cancer.

## Materials and methods

### Data and clinical specimens

The RNAseq data related to LINC02086 in patients with breast cancer were obtained from Gene Expression Profiling Interactive Analysis (GEPIA) database. A total of 43 paired cancer and adjacent-normal specimens were collected from patients receiving surgery at hospital. Our research was approved by the independent ethics committee of Bengbu Medical College Ethics Committee (approval no. 20210625080) and was in accordance with the Declaration of Helsinki.

### Cell culture and transfection

MCF-7, MDA-MB-231, ZR751, and SKBR3 (Human breast cancer cells) and MCF-10A (normal human breast cell) were obtained from the American Type Culture Collection (ATCC, Manassas, USA). Forsubcellular fractionation, cytoplasmic or nuclear RNA was isolated as previously reported [15].

The shRNAs targeting LINC02086 were cloned into a pLKO.1 vector and packaged as lentivirus. For LINC02086 overexpression, the coding sequence was cloned into pLVX-Puro plasmids (Clontech, USA). The miRNAs used in the study (Genepharm Technologies, Shanghai, China) were listed as follows: miR-6757-5p mimic (5′-AGUAGGACCGGAGGGUAGGGAU-3′), miR-6757-5p inhibitor (5′-UCAUCCUGGCCUCCCAUCCCUA-3′), and negative control (5′-UGAGC AAGGGCGAGGAGCUGUUC-3′).Transfection was performed using Lipofectamine 2000 reagent.

### Cell counting kit (CCK)-8 assay and cell apoptosis detection

Briefly, cells (3 × 10^3^ cells/well) in 96-well plates were incubated with CCK-8 solution (10 µL / cell) for 1 h. A microplate reader was used to test cell viability (optical density = 450 nm) [16].

Cell apoptosis was detected by flow cytometry as previously reported [17]. Cells in a 6-well plate (3 × 10^5^ per well) were incubated with 5 μL Annexin-V-FITC for 15 min and 5 μl propidium iodide for 15 min, all from Beyotime Biotechnology (Shanghai, China), and finally, examined by a CytoFLEX flow cytometer (Beckman Coulter, USA).

### Luciferase reporter assay

As previously reported [18], briefly, LINC02086 containing miR-6757-5pcomplementary sequence or EPHA2 3′-UTR sequence was cloned into pmirGLO firefly luciferase reportervector (Promega). For LINC02086 luciferase reporter assay, cells were transfected with miR-6757-5p mimic and pmirGLO-LINC02086-WT (pmirGLO-EPHA2 3′-UTR-WT) or pmirGLO-LINC02086-MUT (pGL3- pmirGLO-EPHA2 3′-UTR-MUT) and pRL-TK vector (Promega) expressing the renilla luciferase. At 48-h, firefly and *Renilla*luciferase activities were measured by the dual-luciferase assay kit (Promega).

### RNA pull-down assay

RNA pull-down assay was implemented as previously reported [19]. Briefly, bio-labeled probe of LINC02086 or control probe (Sangon, Shanghai, China) were transfected into cells. Cell lysates were incubated with Streptavidin-Dyna beads with RNA separation, followed by enrichment of miR-6757-5p by polymerasechainreaction (PCR).

### Quantitative reverse transcriptase polymerasechainreaction (qRT-PCR)

As previouslyreported[20], qRT-PCR wasperformedusing SYBR green PCR master mix (AppliedBiosystems, Foster, CA, USA) on an ABI 9700 real-time PCR system (AppliedBiosystems). The primersused for PCR were as follows: LINC02086-F: 5’-TCCCTTGGAGGTATTGAC-3’; LINC02086-R: 5’-CTCAGAAC AACCGATGAC-3’; EPHA2-F: 5’-GACTACGGCACCAACTTCCA-3’; EPHA2-R: 5’-CTGACGGTGATCTCATCGGG-3’; GAPDH-F: 5’-AATCCCATCACCATC TTC-3’; GAPDH-R: 5’-AGGCTGTTGTCATACTTC-3’. For test miRNA expression, the primersused for PCR were as follows: miR-6757-5p-F: 5’-CGTAGGGATGGGAGGCCA-3’; miR-6757-5p-R: 5’-AGTGCAGGGTCCG AGGTATT-3’; U6-F: 5’-CTCGCTTCGGCAGCACA-3’; U6-R: 5’-AACGCTTCA CGAATTTGCGT-3’.

### Western blot analysis

After SDS-PAGE gel separation, proteins were transferred onto nitrocellulose membranes, which were then incubated with primary antibodies against EPHA2 (ab273118; Abcam) and GAPDH (66,009–1-Ig; Proteintech), followed by HRP-conjugated secondary antibodies (ZB-2305; ZSGB-BIO) [21].

### Xenograft experiments

Mice (4 to 5-week old, *n* = 6 per group) were purchased from Shanghai Laboratory Animal Company (Shanghai, China), and the ethical approvalto perform animal experiments have obtained with No.20210625080 from Bengbu Medical College. MCF-7 cells (5 × 10^6^) transduced with pLKO.1-LINC02086-shRNA or pLKO.1-shNC were subcutaneously injected into the flank regions of mice. Tumor volume was measured at different time points. After 35 days, tumors were harvested from the sacrificed mice. Tumor tissue samples were subjected to TUNEL (Terminal deoxynucleotidyl transferase dUTP Nick-End Labeling) analyses (Roche, Indianapolis, IN, USA). Laboratory experimentation abided by the animal ethics guidelines of Hospital.

### Statistical analysis

All statistical analyses on quantitative data (mean ± SD) were done on GraphPad Prism 8.4.2 (GraphPad Software, San Diego, CA, USA). Overall survival (OS) was determined by Kaplan–Meier survival analysis and log-rank test. Comparison between different groups was performed with ANOVA test or Student’s t test, with *p*-value < 0.05 as threshold of significance.

## Results

### LINC02086 was up-regulated in breast cancer and predicted unfavorable outcome

**Fig. 1:**
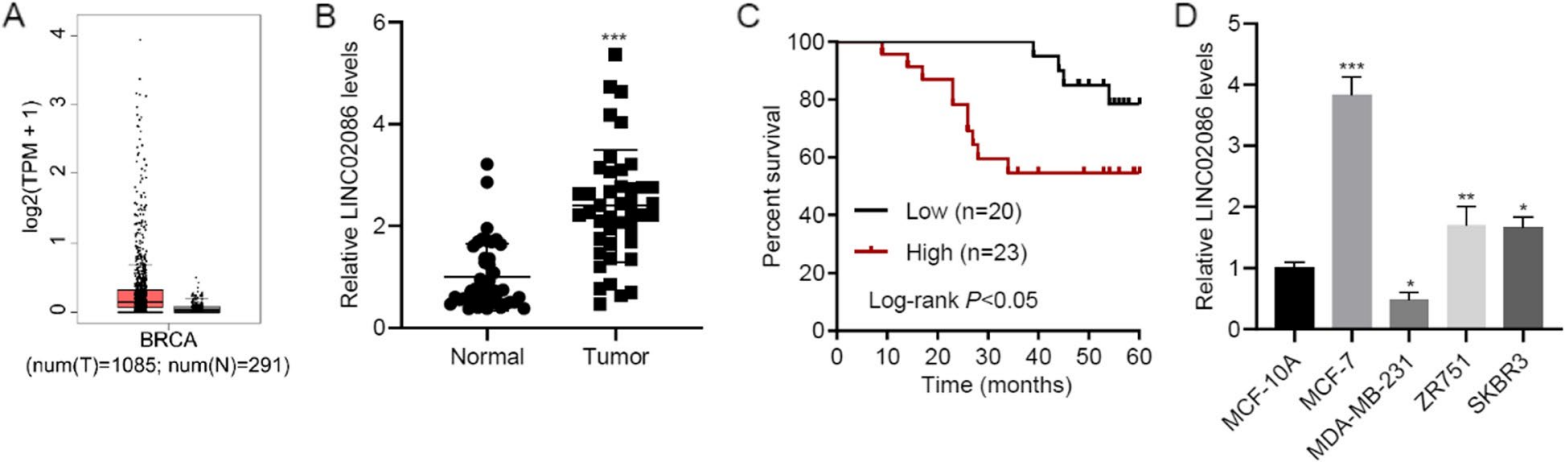
LINC02086 is up-regulated in breast cancer and associated with prognosis. A expression data of LINC02086 downloaded from GEPIA database; B, LINC02086 levels in 43 paired cancer specimens and adjacent-normal specimens; C, survival time of patients with high and low LINC02086 expression; D, LINC02086 levels in MCF-10A,MCF-7, MDA-MB-231, ZR751, and SKBR3 cells. *P < 0.05, **P < 0.01, ***P < 0.001 *vs.* normal or MCF-10A

LINC02086 expression was up-regulated in breast cancer tissue using RNA-seq data of GEPIA database (Fig. 1A). Using qRT-PCR, LINC02086 expression was found to be elevated in 43 cancer specimens compared to adjacent-normal specimens from patients (*p*-value < 0.001, Fig. 1B). Patients with high LINC02086 expression survived shorter than patients with low LINC02086 expression according to the percent survival of patients (Log-rank *P* < 0.05, Fig. 1C). LINC02086 up-regulation was consistently observed in MCF-7 cells (Log-rank *P* < 0.001), ZR751 cells (Log-rank *P* < 0.01) and SKBR3 cells (Log-rank *P* < 0.05) relative to MCF-10A cells (Fig. 1D). Besides, as shown in Table 1, LINC02086 expression was significantly related to tumor stage (*p*-value < 0.0165), lymph node status (*p*-value < 0.0480), estrogen receptor (ER) status (*p*-value < 0.0035), progesterone receptor (PR) status (*p*-value < 0.0246) and human epidermal growth factor receptor 2 (HER2) status (*p*-value < 0.0118).

**Table 1:** Relationship between LINC02086 expression level and clinicopathological parameters of breast cancer

Variable	LINC02086		
	Low (*n* = 20)	High (*n* = 23)	*P* value
Age (years)			0.1582
≥ 58	13	10	
< 58	7	13	
Histological type			0.2363
Ductal	12	19	
Lobular	5	3	
Other	3	1	
Tumor site			0.9202
Left	11	13	
Right	9	10	
AJCC stage			0.0759
I	5	2	
II	13	11	
III	2	8	
IV	0	2	
Tumor stage			0.0165
T1	9	2	
T2	10	13	
T3	1	6	
T4	0	2	
Lymph node status			0.0480
Metastasis	7	15	
No metastasis	13	8	
ER status			0.0035
Positive	5	16	
Negative	15	7	
PR status			0.0246
Positive	8	17	
Negative	12	6	
HER2 status			0.0118
Positive	5	20	
Negative	15	13	

*ER* Estrogen receptor, *PR* Progesterone receptor, *HER2* Human epidermal growth factor receptor type 2. Differences between groups were done by the Chi-square test

### LINC02086 silencing decreased cell viability, promoted cell apoptosis and suppressed tumor growth

**Fig. 2:**
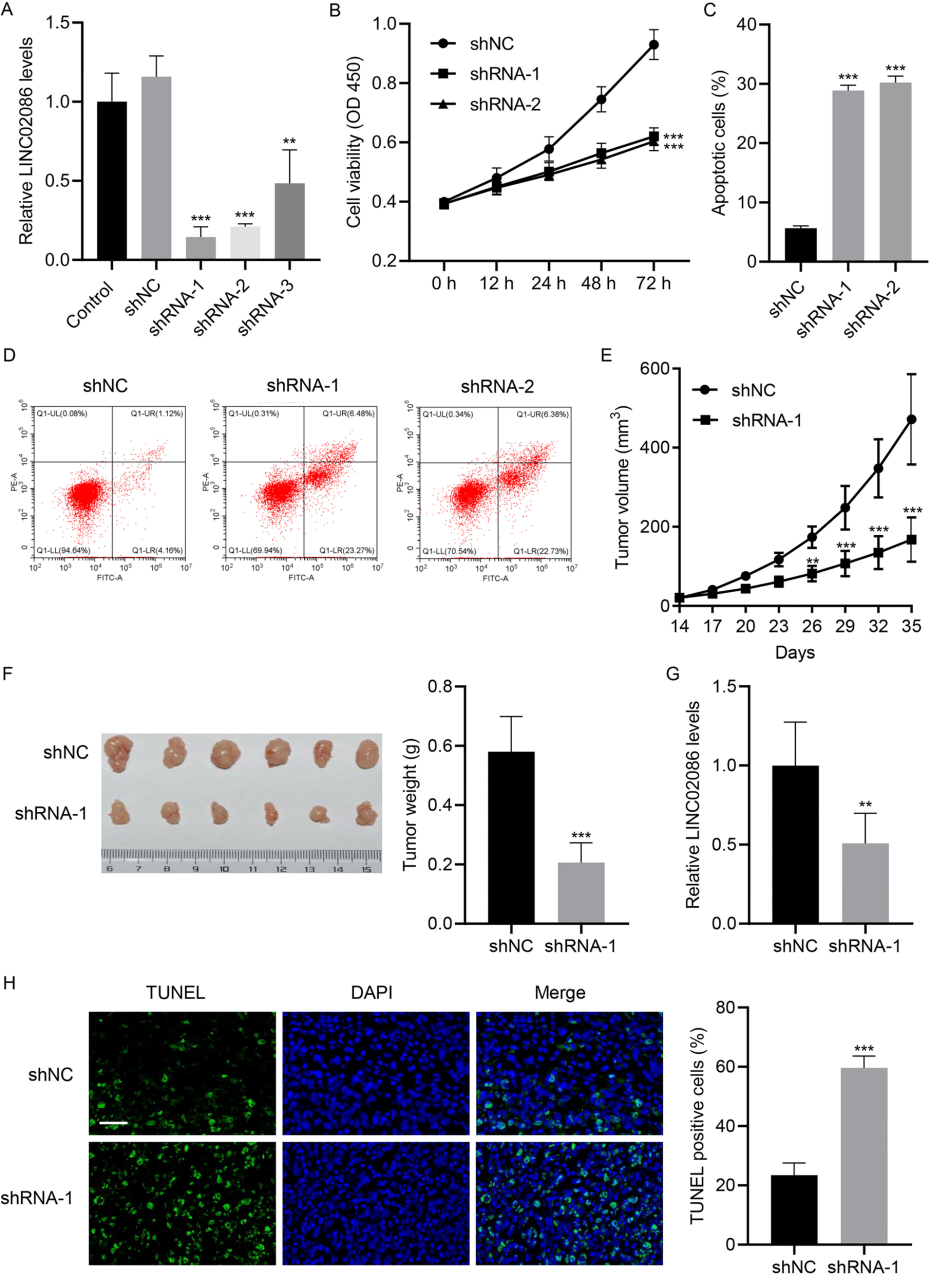
LINC02086 knockdown decreases cell viability, promotes cell apoptosis and inhibits tumor growth. A, LINC02086 knockdown by shRNAs transfection in MCF-7 cells; B, cell viability of MCF-7 cells transfected with shRNA-1, 2 at different time points; C-D, percentages of apoptotic cells detected by flow cytometry; E, tumor volume of mice models at different time points; F, tumor weight of mice models at day 35; G, LINC02086 levels in tumors harvested from mice models; H, TUNEL positive cells on tumor tissue samples harvested from mice models. Scale bar = 50 μm. **P < 0.01, ***P < 0.001 *vs*. shNC

For LINC02086 knockdown, MCF-7 cells were transfected with shRNA-1, -2, -3 targeting LINC02086. As shown in Fig. 2A-B, shRNA-1 and 2 transfections led to successful LINC02086 knockdown (*p*-value < 0.001) and a 40% decrease in cell viability (*p*-value < 0.001) at 72 h. LINC02086 silencing resulted in remarkable increases in percentages of apoptotic cells (Fig. 2C-D, *p*-value < 0.001). Furthermore, shRNA-1-transfected MCF-7 cells were injected into mice to establish xenograft animal models. Tumor volume at 26, 29, 32 and 35 days, and tumor weight at 35 days were decreased in mice models carrying shRNA-1 compared to control models (Fig. 2E-F, *p*-value < 0.001). Moreover, successful LINC02086 silencing by shRNA-1 (Fig. 2G, p-value < 0.01) caused increases in TUNEL positive cells (*p*-value < 0.001, Fig. 2H).

We further overexpressed LINC02086 in MDA-MB-231 cells by lentivirus-mediated transfection (Figure S1A). As a consequence, cell viability was increased and cell apoptosis was suppressed (Figure S1B-C). These results collectively suggest an oncogenic role played by LINC02086 in breast cancer.

### LINC02086 interacted with and negatively correlated with miR-6757-5p

**Fig. 3:**
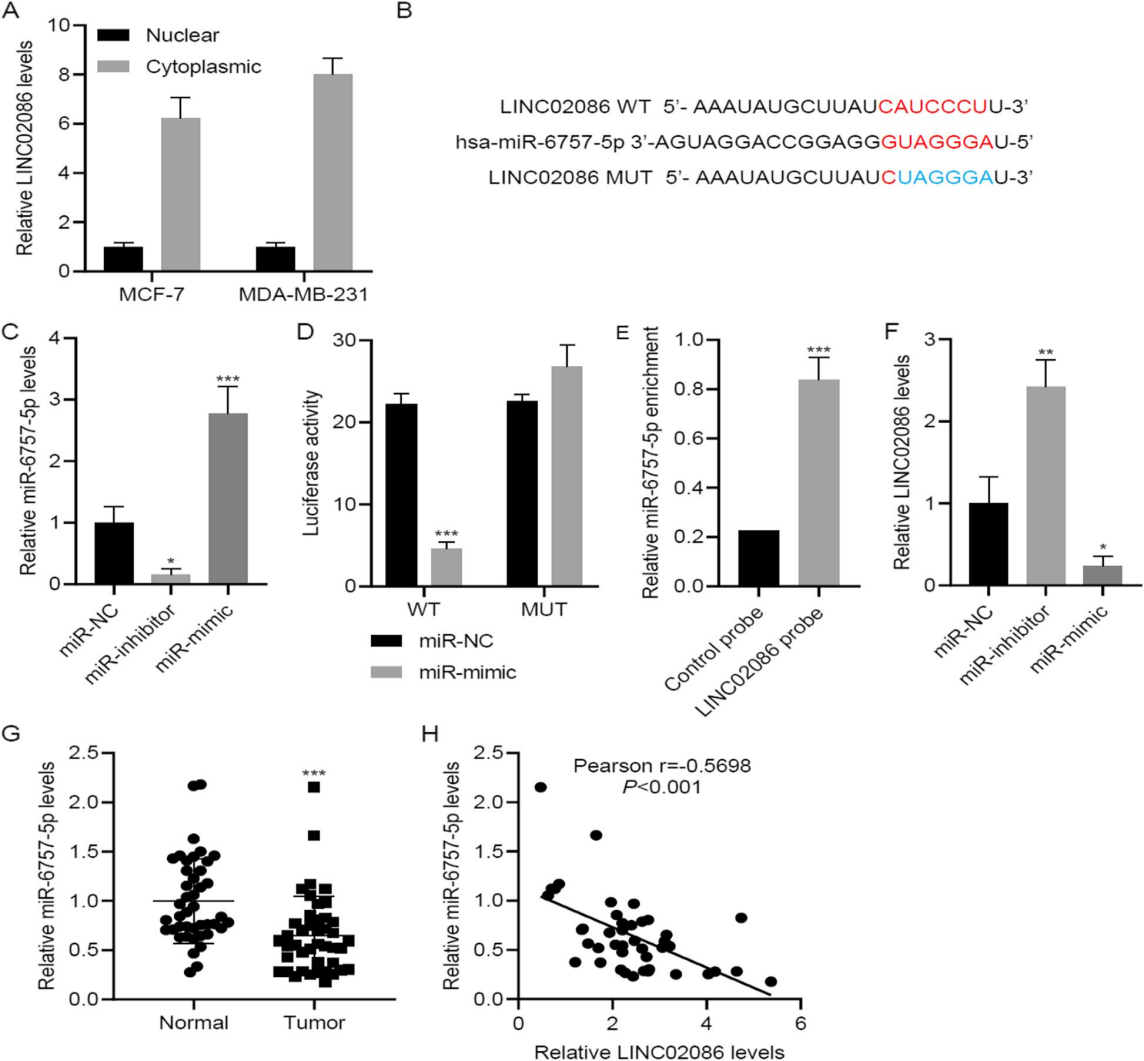
LINC02086 interacts with and negatively correlates with miR-6757-5p. A cytoplasmic/nuclear fractionation of LINC02086 in MCF-7 and MDA-MB-231 cells; B, the binding site between LINC02086 and miR-6757-5p; C, LINC02086 levels in MCF-7 cells transfected with miR-inhibitor or miR-mimic; D, luciferase reporter activity in MCF-7 cells transfected with miR-mimic and wild type or mutant LINC02086. E, miR-6757-5p enrichment with LINC02086 detected by RNA pull down assay. F, LINC02086 levels in MCF-7 cells transfected with miR-inhibitor or miR-mimic; G, miR-6757-5p levels in 43 paired cancer specimens and adjacent-normal specimens; H, correlations of miR-6757-5p levels with LINC02086 levels in cancer specimens. *P < 0.05, **P < 0.01, ***P < 0.001 *vs*. miR-NC, control probe or normal

In either MCF-7 cells or MDA-MB-231 cells, LINC02086 was predominately distributed in cytoplasm rather than nucleus (Fig. 3A). Moreover, we predicted miR-6757-5p binding site of LINC02086 by bioinformatics software (circinteractome). Sequences of wild type and mutant LINC02086, and miR-6757-5p were shown in Fig. 3B. miR-6757-5p mimic and inhibitor were transfected into MCF-7 cells and resulted in significant increases and decreases in miR-6757-5p levels (*p*-value < 0.001, Fig. 3C). Luciferase assay and RNA pull-down assay were used to explore whether miR-6757-5p binds to LINC02086. Luciferase activity was decreased after co-transfection of wild type LINC02086 and miR-6757-5p mimic, but was recovered by co-transfection of mutant LINC02086 and miR-6757-5p mimic (*p*-value < 0.001, Fig. 3D). It revealed the binding site of LINC02086 by miR-6757-5p. Besides, miR-6757-5p enrichment in biotin-labeled LINC02086 confirmed LINC02086 binding to miR-6757-5p (*p*-value < 0.001, Fig. 3E). Transfections of miR-6757-5p inhibitor and mimic promoted and inhibited LINC02086 expression, respectively (*p*-value < 0.05, Fig. 3F). In the patients cohort, miR-6757-5p level was decreased in cancer tissue samples compared to normal tissue samples (*p*-value < 0.001, Fig. 3G), and was negatively correlated with LINC02086 level (Pearson *r* = -0.5698, *P* < 0.001, Fig. 3H).

### miR-6757-5p inhibition compromised the effects of LINC02086 silencing on cell viability and apoptosis

**Fig. 4:**
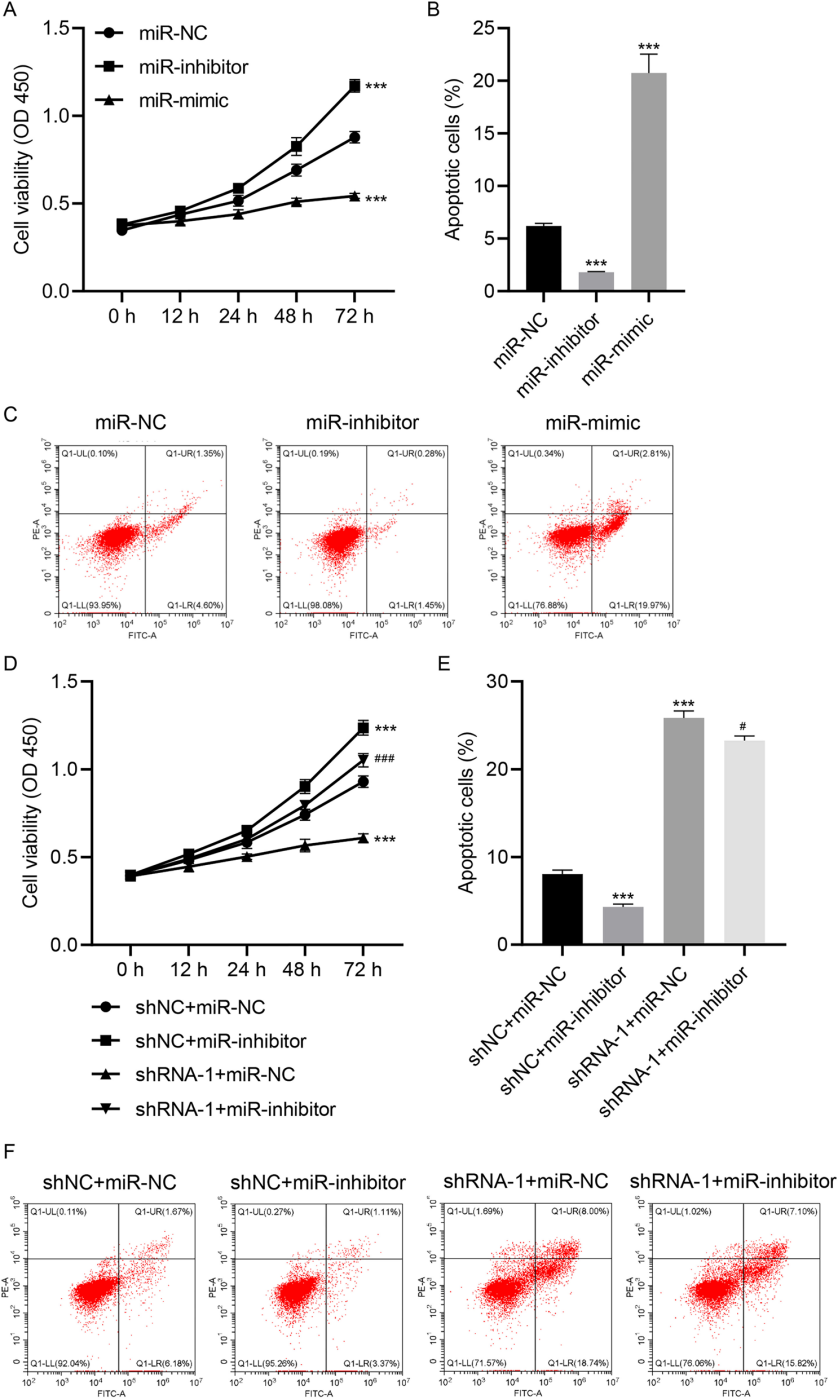
miR-6757-5p inhibitor alleviates the effects of LINC02086 silencing on cell viability and apoptosis. miR-6757-5p inhibitor or mimic is transfected into MCF-7 cells. A, cell viability of MCF-7 cells; B-C, percentages of apoptotic cells detected by flow cytometry. MCF-7 cells are co-transfected with shRNA-1/shNC and miR-inhibitor/miR-NC. D-F cell viability and percentages of apoptotic cells.***P < 0.001 *vs.*miR-NC or shNC + miR-NC. #*P* < 0.05, ###*P* < 0.001 *vs*. shRNA-1 + miR-NC

miR-6757-5p inhibitor and mimic were transfected into MCF-7 cells, respectively. miR-6757-5p inhibitor increased cell viability and decreased apoptotic cells, whereas miR-6757-5p mimic treatment caused opposite effects (*p*-value < 0.001, Fig. 4A-C). As mentioned above, LINC02086 knockdown by shRNA-1 decreased cell viability and promoted cell apoptosis, which were reversed by co-transfection with miR-6757-5p inhibitor (*p*-value < 0.05, Fig. 4D-F).

### LINC02086 promoted EPHA2 expression through down-regulating miR-6757 -5p

Previous studies demonstrate that ephrin type-A receptor 2 (EPHA2) is highly expressed in breast cancer and acts as a key oncogenic tyrosine kinase in tumorigenesis [22, 23]. Therefore, we conducted experiments to test whether LINC02086 and miR-6757-5p affected EPHA2 expression. Silencing LINC02086 by shRNA-1, or -2 transfection into MCF-7 cells gave rise to an obvious decrease in EPHA2 at mRNA and protein levels (p-value < 0.001, Figure S2A-B). Conversely, LINC02086 overexpression in MDA-MB-231 cells promoted EPHA2 at mRNA and protein levels (*p*-value < 0.001, Figure S2C-D).

**Fig. 5:**
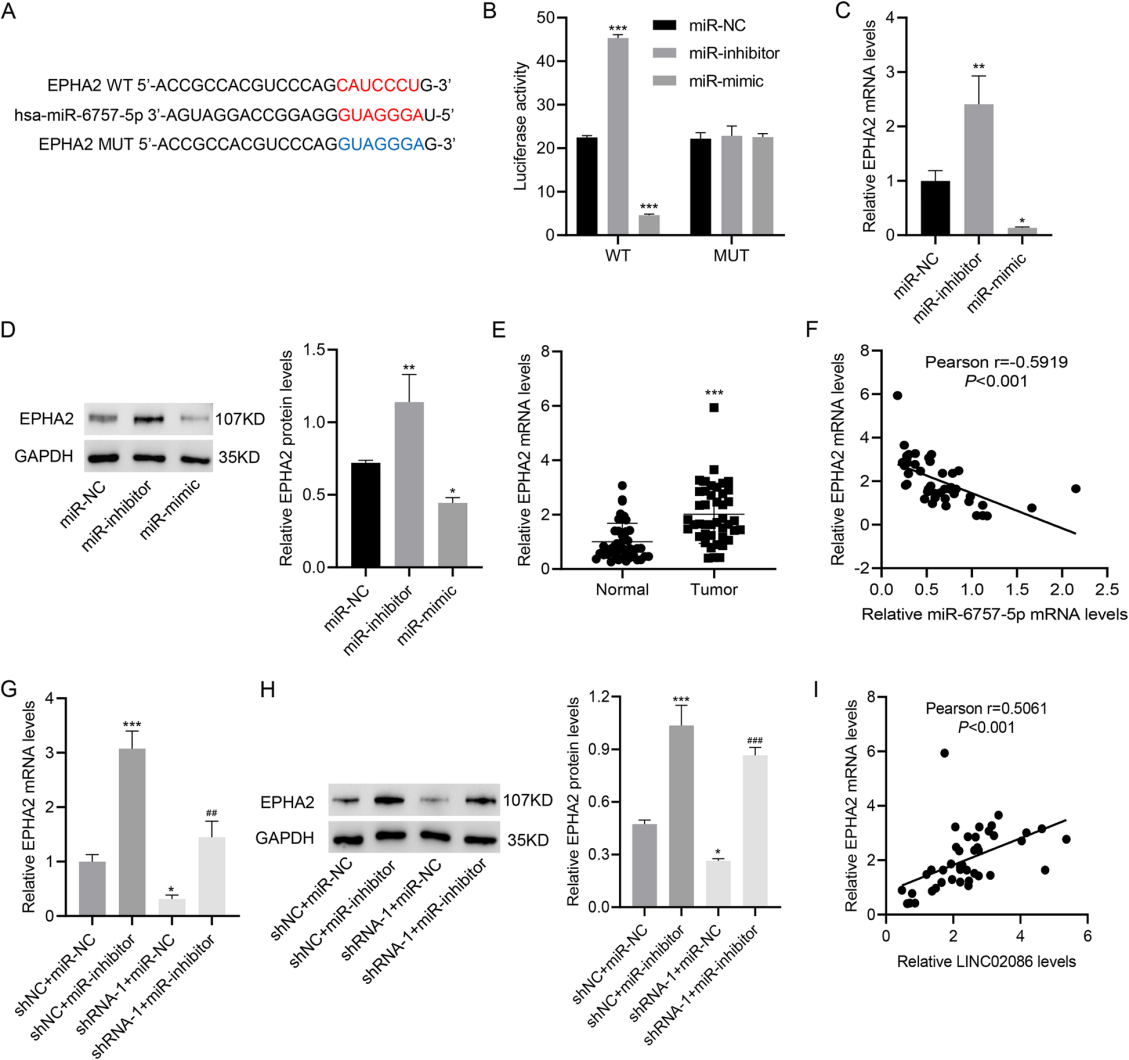
LINC02086 promotes EPHA2 expression via miR-6757-5p. A, the binding site between miR-6757-5p and EPHA2; B, luciferase reporter activity in MCF-7 cells transfected withmiR-mimic/miR-inhibitor and wild type or mutant LINC02086; C-D, EPHA2 mRNA and protein levels in MCF-7 cells transfected with miR-mimic or miR-inhibitor; E, EPHA2 mRNA levels in paired cancer specimens and adjacent-normal specimens; F, correlations between EPHA2 mRNA levels and miR-6757-5p mRNA levels; G-H, EPHA2 mRNA and protein levels in MCF-7 cells co-transfected with shRNA-1/shNC and miR-inhibitor/miR-NC; I, correlations between EPHA2 mRNA levels and LINC02086 levels. *P < 0.05, **P < 0.01, ***P < 0.001 *vs.*miR-NC, normal or shNC + miR-NC. ##*P* < 0.01, ###*P* < 0.001 *vs.* shRNA-1 + miR-NC

The binding site between EPHA2 and miR-6757-5p was analyzed using bioinformatics software (circinteractome) (Fig. 5A). miR-6757-5p inhibitor and mimic were transfected into MCF-7 cells, respectively. Luciferase activity was not significantly changed as a result of mutant EPHA2 with miR-6757-5p mimic or inhibitor (Fig. 5B). EPHA2 expression was up-regulated (*p*-value < 0.01) and down-regulated (*p*-value < 0.05) at mRNA and protein level on exposure to the treatment of miR-6757-5p inhibitor and mimic, respectively (Fig. 5C-D). In the patients cohort, EPHA2 mRNA level was significantly elevated in tumor tissue specimens compared to normal specimens (*p*-value < 0.001, Fig. 5E). Correlation analysis showed that EPHA2 mRNA level was negatively correlated with miR-6757-5p mRNA level in patient cohort (*r* = -0.5919, p-value < 0.001, Fig. 5F). LINC02086 knockdown by shRNA-1 transfection led to a decrease in EPHA2 expression at mRNA and protein level, which was counteracted by the treatment of miR-6757-5p inhibitor (*p*-value < 0.001, Fig. 5G-H). EPHA2 mRNA level was positively correlated with LINC02086 level (*r* = 0.5061, *p*-value < 0.001, Fig. 5I).

## Discussion

Deciphering mechanisms and functions of lncRNAs in carcinogenesis lay a foundation towards development of diagnostic and prognostic biomarkers [24]. Emerging studies have reported an increasing number of lncRNAs as critical regulators implicated in occurrence and progression of breast cancer [25, 26]. Our study focused on the uncharacterized LINC02086 in breast cancer. Although there are limited articles revealing the function of LINC02086 in breast cancer, our study observed an upregulation of LINC02086 in RNA-seq data from the GEPIA database, tumor tissue samples, and breast cancer cells. This upregulation predicted a shorter overall survival time for patients with breast cancer. Consistently, emerging studies have shown that LINC02086 has prognostic value in LSCC [11], hepatocellular carcinoma [12] and lung adenocarcinoma [27]. LINC02086 promoted the proliferation, migration, and invasion of LSCC cells, and mediated the upregulation of PFKFB3 and was associated with a poor prognosis and immune tumor infiltration in LUAD. However, lots of function of LINC02086 in cancers remains unknown.

Our study suggested that LINC02086 exhibited oncogenic activities in breast cancer, as evidenced by decreased cell viability, strengthened cell apoptosis and suppressed tumor growth as results from LINC02086 knockdown. This is the first study that reports the pro-tumorigenic role of LINC02086 in breast cancer. A wealth of studies has suggested that ER status, PR status and HER2 status are significant prognostic factors of breast cancer [28, 29]. Using a cohort of 43 breast cancer patients, our study found thatLINC02086 expression was significantly related to ER status, PR status, HER2 status, tumor stage, and lymph node status. It provides additional evidence that LINC02086 is associated with development and prognosis of breast cancer. Previously, it reports that LINC02086 is related to glycolysis and immune in lung adenocarcinoma [27]. Therefore, the following study will demonstrateLINC02086 may promote the progression of breast cancer through influencing glycolysis and immune.

LncRNAs, miRNAs as well as lncRNA-miRNA axes play crucial functional roles in tumorigenesis[30]. Abundant evidences have shown that lncRNAs can act as decoy for miRNA to prevent the degradation of target genes, or serve as scaffolds to facilitate proteins interactions [31, 32]. It has been reported that miR-6757-3p is associated with the progression and prognosis of pulmonary fibrosis [33]. However, there is a lack of information regarding the role of miR-6757 in cancers, including breast cancer. By analyzing43 paired breast cancer and adjacent-normal specimens, our study revealed that miR-6757-5p was downregulated in breast cancer and exhibited a significant negative correlation with LINC02086 expression. Luciferase assay and RNA pull-down assay consistently demonstrated specific interaction between LINC02086 and miR-6757-5p. Furthermore, we found thatmiR-6757-5pactedas a tumor suppressor by reducing cell viability and promoting cell apoptosis. Moreover, inhibition ofmiR-6757-5p counteracted the effects of LINC02086 silencing on cell viability and apoptosis. Collectively, these results provide initial evidence illustrating that miR-6757-5p mediated the pro-tumorigenic effects of LINC02086 in breast cancer.

EPHA2, a member of the Eph kinase family, plays a crucial role in driving breast cancer metastasis [34, 35]. It exhibits high expression levels in breast cancer tissues and shows significant correlations with lymph node metastasis and clinical tumor stage[36]. The emerging recognition of EPHA2's pivotal involvement in critical processes associated with malignant breast cancer, including proliferation, survival and migration, has spurred the development of promising therapeutic strategies centered around inhibiting EPHA2 [14]. Our study provides compelling evidence that LINC02086 positively regulates the expression of EPHA2 and that there is binding between EPHA2 and miR-6757-5p. Moreover, miR-6757-5p negatively regulates the effects of LINC02086 on EPHA2 expression. This study reports for the first time that LINC02086 modulates EPHA2 expression throughmiR-6757-5p in breast cancer. The oncogenic effect of LINC02086 involves the miR-6757-5p/EPHA2 mechanism. Additionally, the angiogenesis and metastasis of breast cancer are mediated by EPHA2 via AMPK signaling [37]. A recent study reveals that Twist1 regulates EPHA2 expression thereby influencing tumor growth and metastasis in basal-like breast cancer [38]. Furthermore, apart from its association with anticancer immune response activation and cell motility promotion [39], it is imperative to further investigate how LINC02086 affects angiogenesis, metastasis, cell motility as well as immune response to enhance our understanding regarding its biological roles in breast cancer. Moreover, this study identifies LINC02086 as a potential therapeutic target, hence future studies should focus on developing inhibitors for targeting LINC02086.

## Conclusion

In conclusion, our study provides in vivo and in vitro evidences that LINC02086 acts as a tumor-promoter, enhancing cell viability and suppressing cell apoptosis in breast cancer through the regulation ofmiR-6757-5p/EPHA2 axis. LINC02086 acts as a competitive endogenous RNA (ceRNA) for miR-6757-5p, thereby modulating EPHA2 expression. These findings significantly contribute to our understanding of the molecular machinery underlying breast cancer carcinogenesis and suggest that LINC02086 could be a promising strategy for personalized therapeutic interventions against breast cancer.

### Supplementary Information

**Additional file 1:** **Figure S1.** LINC02086 overexpression promotes cell viability and prohibits cell apoptosis. A, lentivirus-mediated LINC02086 overexpression in MDA-MB-231 cells; B, cell viability; C, cell apoptosis detected by flow cytometery. ***P<0.001 *vs.* vector.

**Additional file 2.** LINC02086 up-regulates EPHA2 expression. MCF-7 cells are transfected with shRNAs to knockdown LINC02086. A-B, mRNA and protein levels of EPHA2 in MCF-7 cells transfected with shRNAs; C-D, mRNA and protein levels of EPHA2 in LINC02086-overexpressing MDA-MB-231 cells.

**Additional file 3. **

## Data Availability

The datasets used and/or analysed during the current study are available from the corresponding author on reasonable request.
