# Supplementary figures and images for: LINC02086 promotes cell viability and inhibits cell apoptosis in breast cancer by sponging miR-6757-5p and up-regulating EPHA2
Source: World J Surg Oncol. 2023 Nov 27;21:371. doi: 10.1186/s12957-023-03245-w (PMC10680215; doi:10.1186/s12957-023-03245-w)

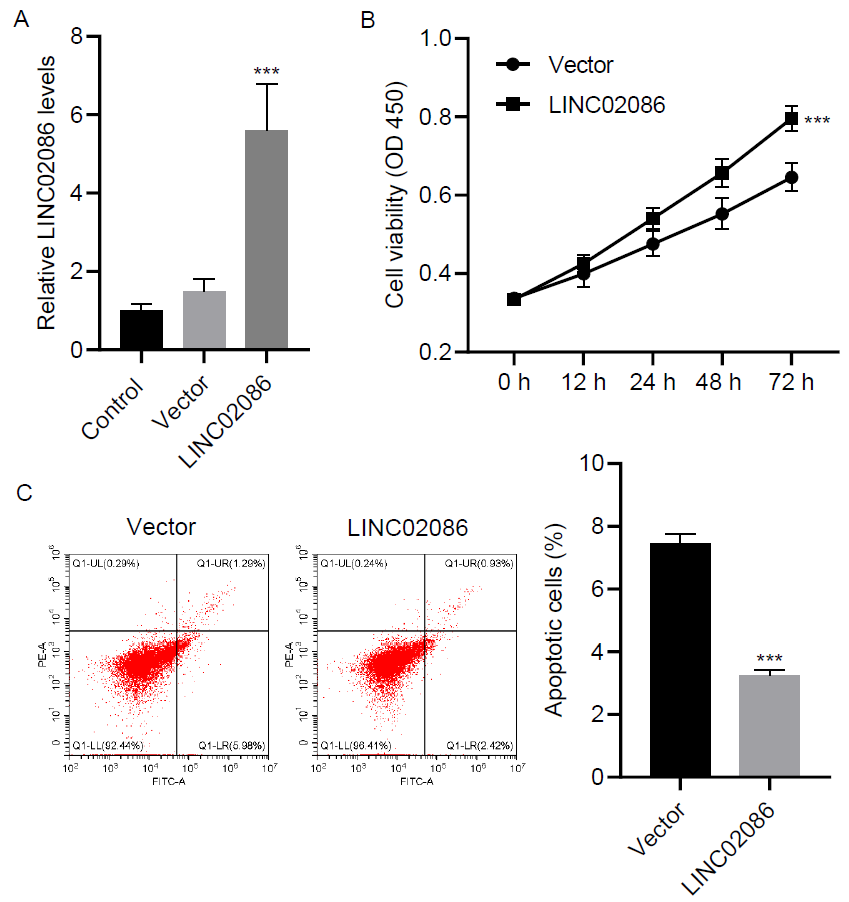

Supplement: Supplementary file 1 — Additional file 1: Figure S1. LINC02086 overexpression promotes cell viability and prohibits cell apoptosis. A, lentivirus-mediated LINC02086 overexpression in MDA-MB-231 cells; B, cell viability; C, cell apoptosis detected by flow cytometery. ***P<0.001 vs. vector. [file 12957_2023_3245_MOESM1_ESM.png]

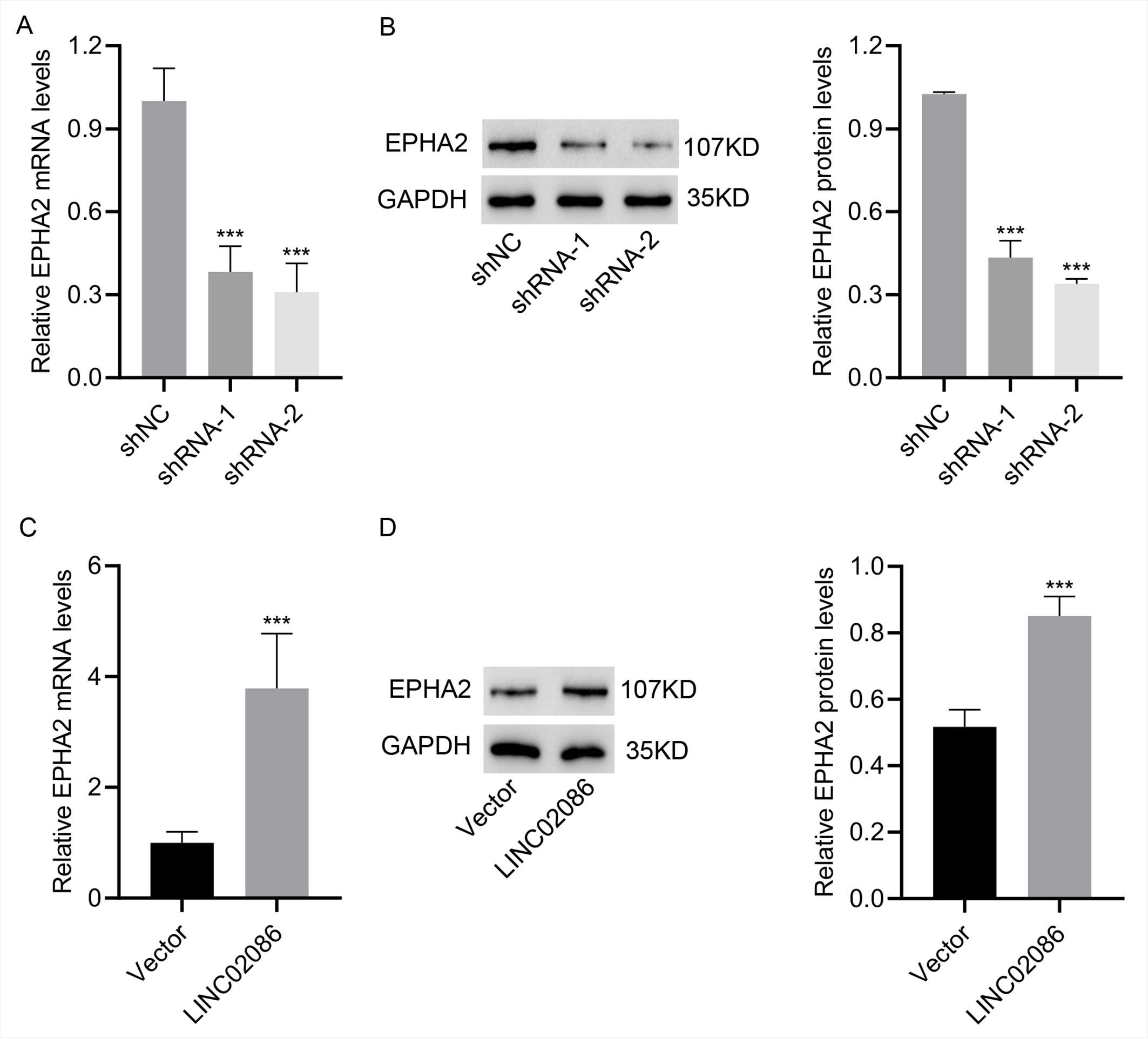

Supplement: Supplementary file 2 — Additional file 2. LINC02086 up-regulates EPHA2 expression. MCF-7 cells are transfected with shRNAs to knockdown LINC02086. A-B, mRNA and protein levels of EPHA2 in MCF-7 cells transfected with shRNAs; C-D, mRNA and protein levels of EPHA2 in LINC02086-overexpressing MDA-MB-231 cells. [file 12957_2023_3245_MOESM2_ESM.tif]

## Slide 1
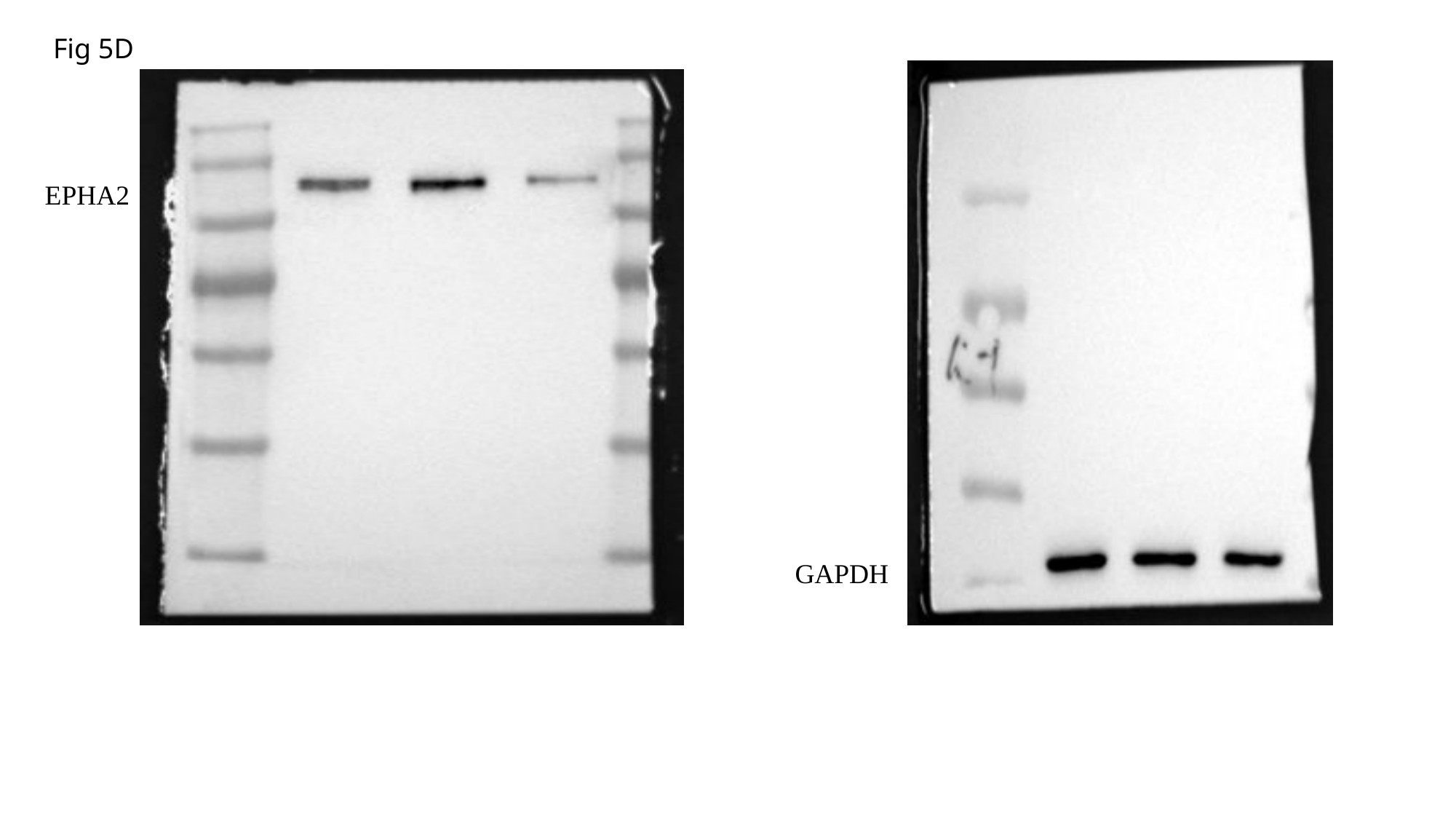

Fig 5D
EPHA2
GAPDH

## Slide 2
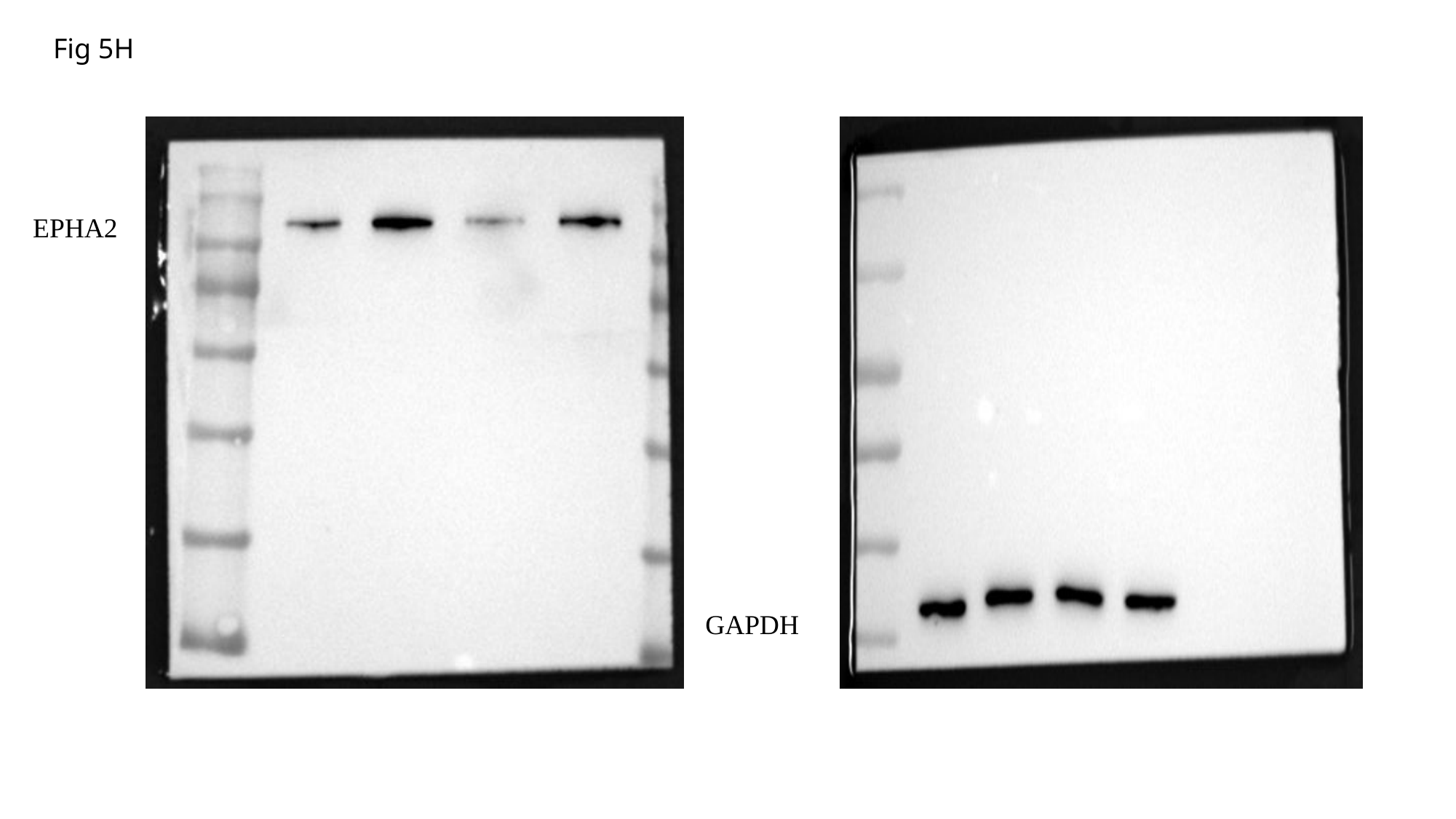

Fig 5H
EPHA2
GAPDH

## Slide 3
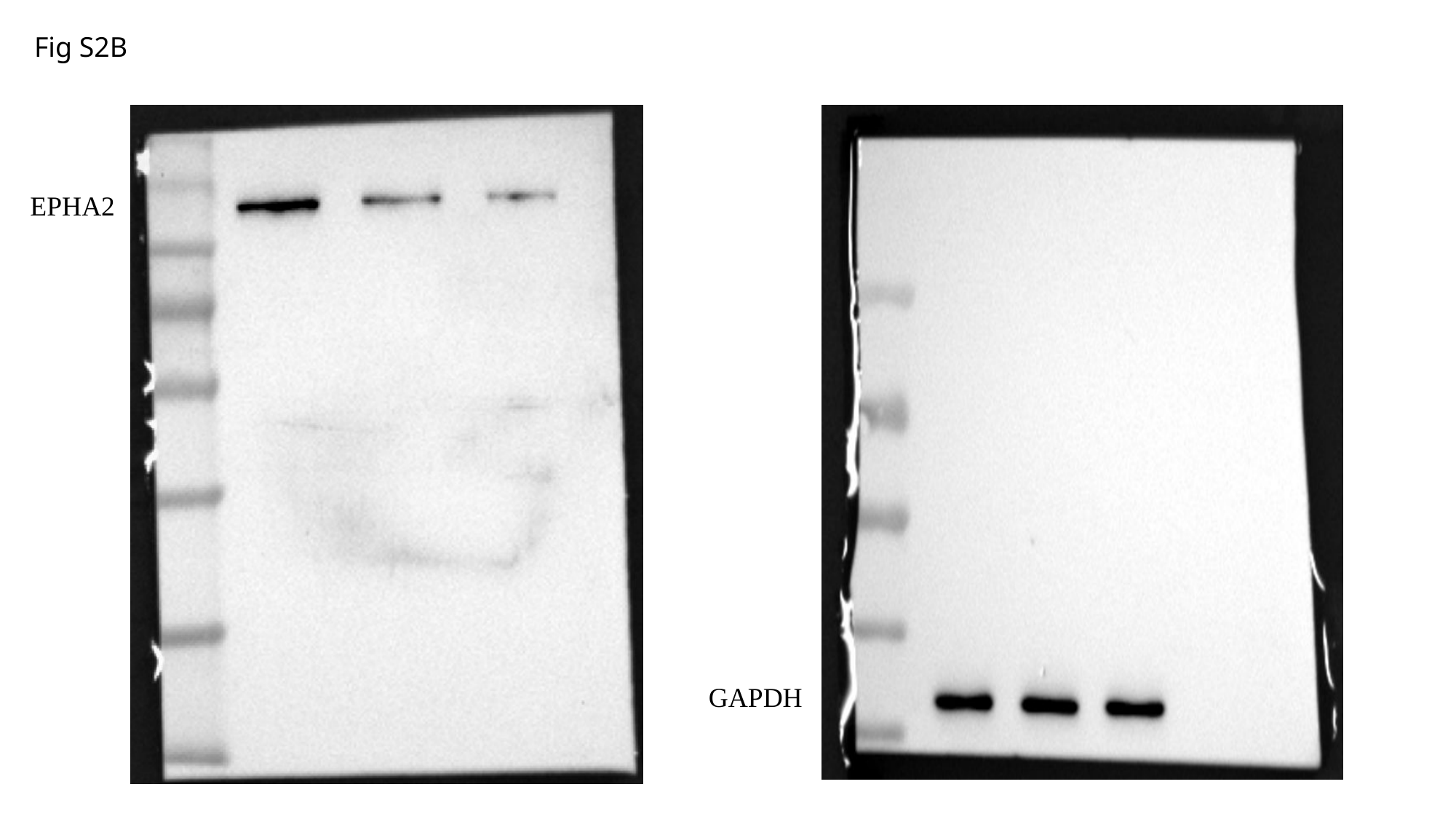

Fig S2B
EPHA2
GAPDH

## Slide 4
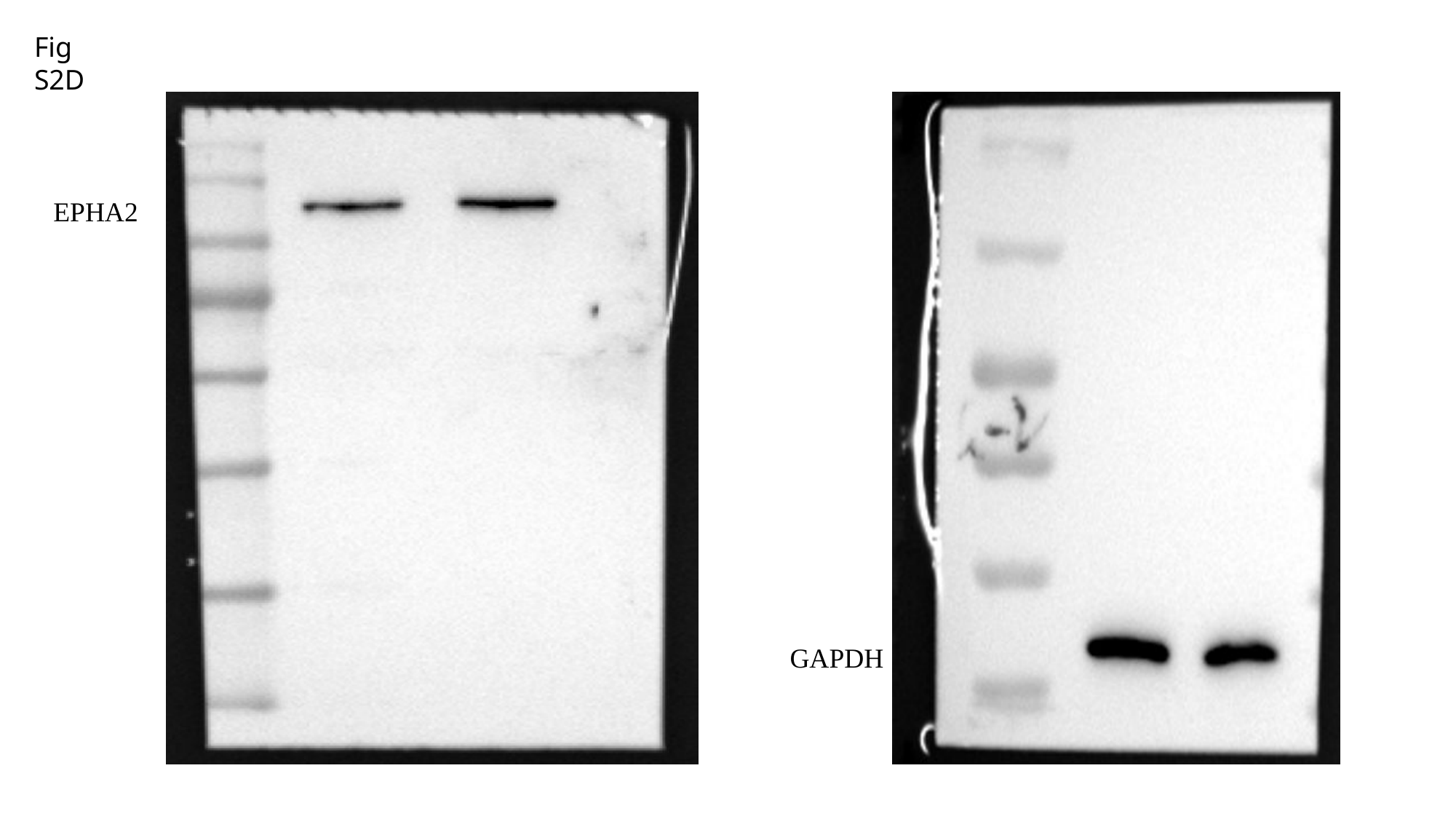

Fig S2D
EPHA2
GAPDH

Supplement: Supplementary file 3 — Additional file 3. [file 12957_2023_3245_MOESM3_ESM.pptx]
